# Socioeconomic Factors and Depressive Symptoms Among Caregivers of Visually Impaired Individuals in South Africa

**DOI:** 10.3390/ijerph23010057

**Published:** 2025-12-31

**Authors:** Dimakatso Given Mashala, Hlupheka Lawrence Sithole, Eric Maimela

**Affiliations:** 1 Department of Optometry, Faculty of Health Sciences, University of Limpopo, Polokwane 0727, South Africa; 2 Department of Public Health, Faculty of Medicine and Health Sciences, Walter Sisulu University, Mthata 5117, South Africa

**Keywords:** informal caregiving, depressive symptoms, socioeconomic status, visual impairment, caregiver well-being, public health, black or African populations, South Africa, depression epidemiology

## Abstract

Caregiving for visually impaired individuals imposes substantial psychological, social, and financial burdens. This study examined depressive symptoms among family caregivers in rural Limpopo, South Africa, and their associations with sociodemographic and socioeconomic factors. A cross-sectional survey was conducted among 253 informal caregivers (response rate: 85.5%). Data on age, gender, marital status, education, employment, income, and socioeconomic status were collected. Depressive symptoms were assessed using the full 20-item Centre for Epidemiologic Studies Depression Scale (CES-D), and associations were analysed using chi-square tests, Cramer’s V, and logistic regression. Moderate-to-severe depressive symptoms were reported by 29.2% of caregivers, with 28.1% experiencing mild-to-moderate symptoms. Male caregivers were less likely to report minimal symptoms (OR = 0.3; 95% CI: 0.12–0.65). Caregivers aged 50–59 years were more likely to report lower depressive symptoms (OR = 1.3). Unmarried caregivers had higher odds of depressive symptoms compared with married caregivers (OR = 2.3). Education was protective: secondary education was associated with lower odds of severe symptoms, while primary education significantly increased risk (OR = 18.1). Lower-income caregivers tended to report higher depressive symptoms. Depressive symptoms among caregivers are influenced by gender, age, marital status, education, and income. Interventions such as psychosocial support, financial assistance, and community-based respite services are essential to reduce caregiver burden.

## 1. Introduction

Visual impairment (VI) affects over 2.2 billion people worldwide, with at least one billion cases being preventable or treatable [1]. The burden of VI is disproportionately high in low- and middle-income countries, particularly in sub-Saharan Africa, where poverty, inadequate access to eye care services, and limited awareness exacerbate the impact of vision loss [2]. Beyond the visual disability itself, VI has profound psychological, social, and economic repercussions for affected individuals and their families. Dependence in activities of daily living, reduced mobility, and challenges in health management often transfer a substantial caregiving burden to informal caregivers. According to the National Alliance for Caregiving and the American Association of Retired Persons [3], approximately 43.5 million caregivers in the United States provide unpaid care to older adults, with 34.2 million caring for individuals aged 50 years and older. In the African context, the Africa Care Economy Index, a tool that assesses countries’ performance in recognising, supporting, and redistributing care work, reveals that females undertake 70% of caregiving. In comparison, the remaining 30% is provided by individuals outside the family and by volunteers who are often minimally compensated [4]. This largely unpaid and under-recognised labour underpins informal caregiving.

Informal caregiving represents an essential but often invisible component of healthcare systems, particularly in regions where formal support structures are weak or unavailable. Caregiver burden encompasses the emotional, physical, social, and financial strain that arises from providing sustained care to individuals with chronic or disabling conditions [5,6]. In resource-limited contexts such as South Africa, where formal respite services are scarce, informal caregivers, usually family members, play a pivotal role in maintaining the well-being and rehabilitation of visually impaired persons [7,8,9,10,11]. However, the psychological toll of caregiving, particularly in the form of depressive symptoms, often goes unnoticed.

Previous research has highlighted the mental health consequences of caregiving in chronic illnesses such as dementia and stroke [5,12], yet limited studies have examined caregivers of visually impaired individuals, especially within the African context. Limpopo Province, one of South Africa’s least resourced regions, faces high unemployment, persistent poverty, and limited health infrastructure [11]. These conditions amplify caregivers’ emotional and economic vulnerabilities. Moreover, cultural expectations that caregiving is predominantly a female responsibility may obscure the mental health challenges faced by male caregivers, who increasingly participate in care roles [13,14,15]. Understanding how sociodemographic and socioeconomic factors contribute to caregiver depressive symptoms is critical to developing appropriate interventions and mental health support strategies.

From a public health perspective, caregiver burden has profound implications. It contributes to burnout, reduced productivity, psychological morbidity, and diminished quality of life for both caregivers and care recipients [16,17,18]. Addressing caregiver mental health is therefore vital to ensuring sustainable eye-care services and holistic patient rehabilitation. This study aimed to examine the relationship between caregivers’ sociodemographic and socioeconomic characteristics and depressive symptoms among informal caregivers of visually impaired individuals attending a tertiary-academic eye clinic in Limpopo Province, South Africa. The study hypothesised that lower socioeconomic status, female gender, and unemployment would be associated with higher depressive symptoms.

## 2. Materials and Methods

### 2.1. Study Design

A cross-sectional, quantitative survey design was employed to examine the relationship between caregivers’ sociodemographic and socioeconomic factors and their levels of depressive symptoms. This design enabled the researchers to collect data from participants at a single point in time and identify statistical associations between variables.

### 2.2. Study Setting and Population

The study was conducted at Mankweng Tertiary Academic Hospital, located in Limpopo Province, South Africa. The hospital serves a predominantly rural area and caters mainly to a Black African population. In this study, interviews were conducted with caregivers responsible for individuals living with visual impairment. This approach enabled a clear contextualisation of the findings within the specific sociocultural and healthcare realities of the South African population served by the facility. The population comprised unpaid adult caregivers (aged 18 years and above) who provided informal care to visually impaired individuals attending the hospital’s outpatient eye clinic. Eligible participants included spouses, adult children, other relatives, or close friends who had been caring for the visually impaired person for at least one year in a home setting (non-institutionalised). Eligibility of care recipients was verified from clinical records using the World Health Organization criterion for visual impairment, defined as visual acuity worse than 6/60 in the better-seeing eye. This verification was further confirmed during the patients’ clinic visits.

### 2.3. Sampling and Sample Size

A non-probability purposive sampling technique was employed to recruit participants who met the inclusion criteria. The selected participants were caregivers directly involved in the provision of care to visually impaired individuals, as they had relevant caregiving experience. The chosen sampling method enabled the study to capture in-depth, context-specific information about depressive symptoms among caregivers. The minimum required sample size (*n* = 296) was calculated using Cochran’s formula, based on an estimated prevalence of 26.4% for visual impairment [19], a 5% margin of error, and a 95% confidence interval. A total of 253 participants were successfully enrolled, yielding a response rate of 85.5%. Although slightly below the calculated sample size, this sample was deemed sufficient to maintain statistical power, given the observed effect sizes and response distribution.

### 2.4. Data Collection

Primary data were collected from caregivers using a structured, interviewer-administered questionnaire over three months by the researcher (D.G.), who was fluent in the local language to ensure accurate communication. Depressive symptoms were assessed using the 20-item Centre for Epidemiologic Studies Depression Scale (CES-D), scored from 0 (“Rarely or None of the Time”) to 3 (“Most or Almost All the Time”), with total scores ranging from 0 to 60. In South Africa, the CES-D has shown reliability of 0.90 among students [20] and 0.92–0.93 among schoolteachers [21], supporting its applicability despite limited validation among caregivers. The 10-item CES-D (CES-D-10) has also been validated in Zulu, Xhosa, and Afrikaans populations (α = 0.69–0.89; AUC = 0.81–0.94) [22]. The English CES-D was translated and back-translated, with discrepancies resolved by a language expert. Sociodemographic data included age, gender, marital status, education, employment, and income.

### 2.5. Data Analysis

Socioeconomic status was categorised according to the Modified Kuppuswamy Socioeconomic Scale (upper, upper-middle, lower-middle, upper-lower, and lower class). CES-D scores were classified as: little or no depression (0–9), mild depression (10–15), moderate depression (16–24), and severe depression (≥25) [19]. Data were entered into Microsoft Excel and analysed using SPSS version 29. Missing data were handled using multiple imputation, in which missing values for key variables were estimated from observed data to reduce bias and preserve statistical power. Descriptive statistics (frequencies, percentages, and means) were used to summarise participant characteristics. Independent variables included caregivers’ demographic and socioeconomic factors, while caregiver depressive symptoms (CES-D scores) served as the dependent variable. Categorical variables were examined using chi-square tests, and effect sizes were quantified with Cramer’s V to assess the strength of associations. Univariate linear regression was performed to evaluate the relationship between the continuous dependent and independent variables. Statistical significance was set at *p* < 0.05.

### 2.6. Ethical Considerations

Ethical approval was obtained from the Turfloop Research Ethics Committee of the University of Limpopo (TREC/326/2021:PG, approval date: 8 December 2021). Permission to conduct the study was granted by the Limpopo Provincial Department of Health and by the management of Mankweng Hospital. All participants provided written informed consent before participation. Confidentiality and anonymity were maintained throughout the study by assigning identification codes instead of names. The study adhered to the ethical principles outlined in the Declaration of Helsinki.

## 3. Results

### 3.1. Characteristics of Family Caregivers’ Sociodemographic and Socioeconomic Status for the Visually Impaired

Table 1 presents the association between participants’ sociodemographic and socioeconomic characteristics and gender among family caregivers of the visually impaired as determined through a cross-tabulation. A total of 253 informal caregivers of visually impaired patients participated in the study, representing an 85.5% response rate from the calculated sample of 296. The mean age of the caregivers was 45.11 ± 14.13 years (range: 22–88). Most participants (32.4%) were within the 35–49-year age group, followed by those aged 50–59 years (18.2%). The association between age group and gender was statistically significant (*p* = 0.003; Cramer’s V = 0.251), indicating a moderate association between gender and age distribution. Regarding marital status, 39.1% of caregivers were married, while 52.7% had never married or cohabited. No statistically significant relationship was observed between marital status and gender (*p* = 0.121; Cramer’s V = 0.152), suggesting a weak association. Educational attainment was generally high, with 52.2% of caregivers having completed secondary education and 35.9% holding tertiary qualifications; although this relationship was statistically significant (*p* = 0.034), Cramer’s V = 0.164 indicated only a weak association. Employment status revealed a stronger gender difference, with 65.8% of males and only 25.2% of females employed, a statistically significant relationship (*p* < 0.001; Cramer’s V = 0.382) indicating a moderate-to-strong association between gender and employment status. Income distribution also varied significantly by gender (*p* < 0.001; Cramer’s V = 0.374), suggesting a moderate-to-strong association, with males more represented in higher income brackets and females predominating in lower-income categories. Socioeconomic classification based on the Modified Kuppuswamy Scale showed that 52.6% of participants were in the upper-lower class, 17.8% in the upper-middle class, and 4.7% in the upper class, with a statistically significant association between gender and socioeconomic status (*p* < 0.001; Cramer’s V = 0.329), indicating a moderate association. While gender differences were statistically significant for most sociodemographic and socioeconomic indicators, the strength of these associations, as measured by Cramer’s V, ranged from weak to moderate to strong, with the most pronounced gender disparities observed in employment, income, and socioeconomic status.

### 3.2. Prevalence of Caregivers’ Depressive Symptoms Among the Study Participants

**Figure 1:**
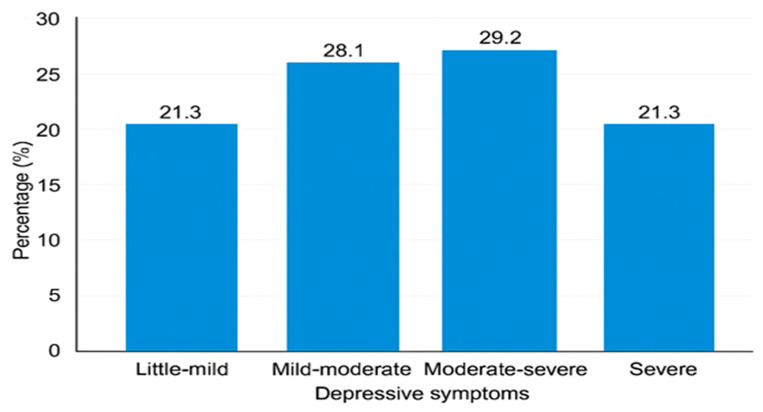
Prevalence of depressive symptoms among caregivers.

Figure 1 presents the prevalence of caregivers’ depressive symptoms. The highest proportion of caregivers (29.2%) experienced moderate-to-severe depressive symptoms, indicating a considerable mental health burden among caregivers. The second highest group (28.1%) reported mild-to-moderate symptoms, suggesting that many caregivers experience some degree of emotional distress even if not clinically severe. Little-to-mild and severe symptoms were reported equally at 21.3% each, reflecting that while some caregivers cope relatively well, a similarly significant proportion experience severe depressive symptoms.

### 3.3. The Mean Scores for Depressive Symptoms with a Standard Deviation Among the Participants

Table 2 presents the mean and standard deviation for the likelihood that caregivers will develop depressive symptoms. The mean scores for individual CES-D items are shown in Table 3. The highest mean scores corresponded to the items “Everything I did was an effort” (1.28 ± 1.107) and “I felt hopeful about the future” (1.27 ± 1.195), suggesting emotional strain coupled with resilience among caregivers. The lowest mean scores were recorded for “People dislike me” (0.65 ± 0.925) and “I could not get going” (0.55 ± 0.870), indicating limited feelings of social rejection or inactivity.

### 3.4. The Distribution of Depressive Symptom Severity Among Caregivers by Gender

**Figure 2:**
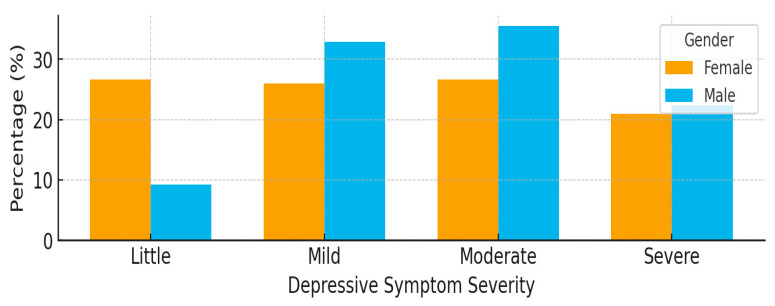
A clustered bar chart illustrating the distribution of depressive symptom categories by gender (*n* = 253).

Figure 2 presents the distribution of depressive symptom severity among caregivers (*n* = 253) by gender. The clustered bar chart illustrates the proportion of caregivers reporting little, mild, moderate, and severe depressive symptoms. Males reported slightly higher proportions of mild and moderate symptoms compared to females. In contrast, severe symptoms were similar between genders, with 22.4% of males and 20.9% of females experiencing severe depressive symptoms (*p* = 0.019), indicating a statistically significant difference in symptom distribution. Age differences were also observed, with caregivers aged 35–49 exhibiting the highest proportion of moderate depressive symptoms (35.4%) compared to those aged 70 and above (18.8%).

### 3.5. Relationship Between Sociodemography and Socioeconomic Factors with Caregiver Depressive Symptoms

Table 3 presents the relationship between sociodemographic and socioeconomic variables and the severity of depressive symptoms among caregivers of the visually impaired. Although depressive symptoms were observed across all age groups, the differences were not statistically significant (*p* = 0.211; Cramer’s V = 0.157), indicating a weak association. Younger adults (22–34 years) showed a higher proportion of moderate depressive symptoms (29.8%), whereas middle-aged caregivers (50–59 years) exhibited more severe symptoms (32.6%). Marital status was not significantly associated with depressive symptoms (*p* = 0.058; Cramer’s V = 0.146), although divorced caregivers (41.7%) and those with deceased partners (44.4%) had higher levels of depression compared to married participants (27.3%). Educational level showed a significant relationship (*p* < 0.001; Cramer’s V = 0.481), with caregivers with no or primary education reporting the highest prevalence of severe symptoms (53.3%), while those with tertiary education reported the lowest prevalence (9.9%). Employment status was also significantly related to depressive symptoms (*p* < 0.001; Cramer’s V = 0.398), with unemployed caregivers exhibiting higher levels of mild-to-moderate depression compared to employed participants. Income demonstrated the strongest association (*p* < 0.001; Cramer’s V = 0.757), as caregivers earning below R812.09 experienced markedly higher rates of moderate-to-severe depressive symptoms, whereas those in the highest income bracket (>R16,221.86) reported the lowest prevalence, highlighting the significant influence of financial hardship on caregivers’ mental health.

### 3.6. Distribution of Depressive Symptom Severity Across Socioeconomic Categories

**Figure 3:**
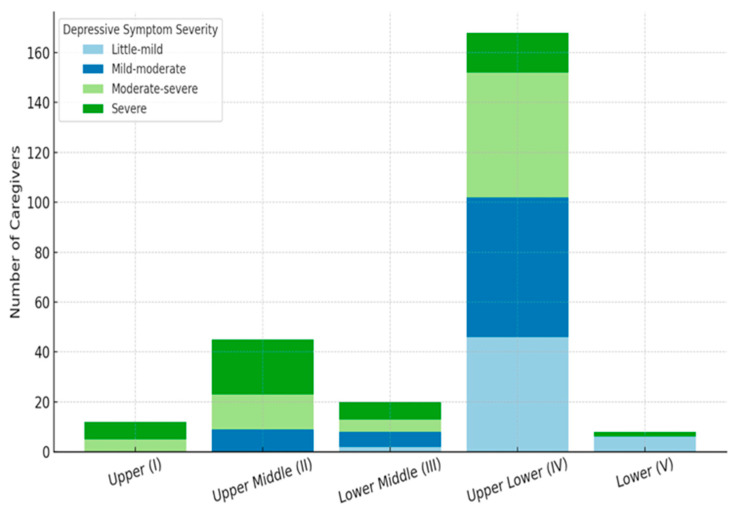
Distribution of depressive symptom severity across socioeconomic classes.

Figure 3 illustrates the distribution of depressive symptom severity among caregivers across different socioeconomic classes. A significant association was observed between socioeconomic status and depressive symptom severity (*p* < 0.001). Caregivers in the upper (I) and upper-middle (II) classes exhibited the highest proportions of severe depressive symptoms (58.3% and 48.9%, respectively), indicating considerable psychological distress despite their higher economic standing. In contrast, caregivers in the upper-lower (IV) class, representing the majority (66.4%), displayed a wider spread of depressive symptoms, with nearly one-third experiencing mild-to-moderate (33.3%) and moderate-to-severe (29.8%) symptoms. The lower-middle (III) group showed a similar trend of moderate symptom distribution, while those in the lower (V) class, although few (3.2%), predominantly reported little-to-mild depressive symptoms (75%). The overall pattern suggests that depressive symptom burden varied significantly by socioeconomic class, with unexpectedly higher severity among caregivers of higher socioeconomic standing.

### 3.7. Univariate Associations Between Sociodemographic, Socioeconomic Factors, and Depressive Symptoms

Table 4 presents the univariate logistic regression analysis of various demographic and socioeconomic factors related to depressive symptomatology among caregivers. Males were less likely than females to experience little to moderate depressive symptoms, with this result being statistically significant (*p* > 0.001); however, they were more likely to experience mild to severe symptoms, though these findings were not statistically significant (*p* > 0.050). Caregivers aged 50–59 years were more likely to have little to mild depressive symptoms, and this association was statistically significant (*p* < 0.050), whereas those aged 35–49 were more likely to experience severe depressive symptoms. However, this was not significant (*p* > 0.050). Participants who had never married were approximately twice as likely to report depressive symptoms compared with married caregivers, and this association was significant (*p* < 0.050). Educational level was strongly associated with depressive symptoms: caregivers with secondary and primary education were more likely to experience mild to moderate depressive symptoms, with statistical significance (*p* < 0.050), whereas secondary-educated participants were less likely to experience severe symptoms (*p* > 0.001) and those with primary education were also less likely to experience severe symptoms (*p* < 0.001). Income showed mixed associations: caregivers earning R8111.24–R16,221.86 were more likely to have mild to moderate depressive symptoms, though this was not statistically significant (*p* > 0.050), while those earning R2432.98–R4055.51 were less likely to have little to mild symptoms (*p* < 0.050) but more likely to experience mild to severe symptoms, though not significantly (*p* > 0.050). Similarly, participants earning R812.10–R2432.97 were more likely to report mild to moderate depressive symptoms, but this association was not significant (*p* > 0.050). Overall, univariate analysis indicates that gender, age, marital status, education, and income are important factors influencing depressive symptom severity, with education and income showing the strongest and most consistent associations.

## 4. Discussion

The present study aimed to investigate the prevalence of depressive symptoms and their association with socioeconomic factors among caregivers of visually impaired individuals in rural Limpopo. The overall prevalence of moderate to severe depressive symptoms was substantial, consistent with previous research [5], which reported a significant mental health burden among caregivers in similar contexts. The findings of the current study underscore the emotional toll of caregiving, particularly in resource-limited settings. It aligns with Braich et al. [12], who reported high depressive symptom prevalence among caregivers of visually impaired individuals in India.

Regarding gender, over a quarter of caregivers in this study were male, despite societal and cultural norms that traditionally designate females as family caregivers [13,14]. This finding is consistent with Lopez–Anuarbe and Kohli [15], who observed an increasing involvement of men in caregiving roles. The emergence of male caregivers suggests that caregiving responsibilities are becoming less gendered and that males are as capable as their female counterparts. In the present study, univariate analyses indicated that male caregivers were less likely to report little depressive symptoms than females (OR = 0.3; 95% CI: 0.12–0.65; *p* > 0.001), but more likely to experience mild to severe depressive symptoms, though these associations were not statistically significant (*p* > 0.050). These findings are consistent with those of Zwar et al. [18] and Ruiz-Lozano et al. [5] and may reflect the greater physical and logistical challenges male caregivers face, including assisting with mobility and navigating public aid services. Male caregivers also tend to have smaller social networks and are less likely to engage with support systems due to traditional masculine norms [15]. Therefore, supportive factors such as family support, opportunities to decompress, and appreciation from care recipients are essential as targeted interventions to strengthen caregivers’ coping and caregiving capacity.

Age was also an important factor influencing depressive symptoms, with adults aged 35–49 more likely to provide care than older adults (75+), consistent with national demographic data [23] and Yakubu and Schutte [24]. Caregivers aged 50–59 were more likely to experience little to mild depressive symptoms (OR = 1.3; 95% CI: 1.0–1.58; *p* < 0.050), whereas caregivers aged 35–49 were more likely to report severe depressive symptoms, though this difference was not statistically significant (*p* > 0.050). These findings suggest that adult caregivers in midlife bear a considerable caregiving burden, highlighting the importance of targeted support programmes, including community health worker-led respite services, to alleviate stress.

Marital status also influenced depressive symptoms. Less than half of caregivers were married, while over half had never married, slightly higher than reported by Sanuade and Boatemaa [25]. This may reflect differences in social support availability, as unmarried caregivers may lack spousal support, increasing vulnerability to depressive symptoms. Caregivers who had never married were about twice as likely to report depressive symptoms compared with married caregivers (OR = 2.3; 95% CI: 1.23–4.30; *p* < 0.050), whereas divorced or widowed caregivers showed no significant association. This finding aligns with previous research that reported that unmarried caregivers have significantly increased odds of a 12-month emotional disorder [26]. Similarly, Provenzano et al. [27] found that unmarried grandparent caregivers had higher depressive symptom scores compared with married caregivers, supporting the elevated depression risk among those not in a marital relationship. The findings of the current study underscore the need for psychosocial, financial, and community-based interventions targeting unmarried caregivers.

Education, a key socioeconomic factor, was strongly associated with depressive symptoms (*p* < 0.001). Most caregivers had secondary education or higher, reflecting South Africa’s high literacy rate (>95%) [21]. Caregivers with secondary education were more likely to report mild to moderate depressive symptoms (OR = 3.9; 95% CI: 1.12–13.65; *p* < 0.050) but less likely to experience severe symptoms (OR = 0.3; 95% CI: 0.10–0.56; *p* = 0.001). Those with primary education were at markedly higher odds of experiencing little (OR = 18.1; 95% CI: 2.36–139.07; *p* < 0.050) and mild to moderate depressive symptoms (OR = 4.0; 95% CI: 1.11–14.29; *p* < 0.050), but less likely to report severe symptoms (*p* < 0.001). These findings suggest that higher education may confer protective benefits via enhanced coping skills and access to resources, while lower education limits awareness of support services and heightens vulnerability to stress [28,29,30]. Unemployment and out-of-pocket hospital expenses further exacerbate depressive symptoms by increasing financial strain.

Income, another socioeconomic factor, also contributed to depressive symptomatology. Caregivers earning R8111.24–R16,221.86 were more likely to have mild to moderate depressive symptoms, though not statistically significant (*p* > 0.050). Those earning R2432.98–R4055.51 were less likely to have little to mild symptoms (*p* < 0.050) but more likely to report mild to severe symptoms (*p* > 0.050). High-income caregivers (>R16,221.87) generally reported lower depressive symptoms, yet they still experienced emotional strain due to employment-related caregiving demands such as missed work and productivity loss [24,31]. These findings indicate that financial resources alone may not fully buffer caregivers from depressive symptoms, highlighting the need for psychosocial support across all income groups.

Integration of univariate odds ratios confirms that socioeconomic factors, specifically education and income, are the strongest predictors of depressive symptom severity. In contrast, gender, age, and marital status exert more variable effects. Male caregivers, midlife adults, unmarried caregivers, and those with lower education or income are at elevated risk for depressive symptoms. These results highlight the need for targeted interventions that include psychosocial support, financial assistance, and community-based respite programs for both traditional female caregivers and the growing population of male caregivers.

## 5. Limitations and Future Directions

This study has several limitations that should be considered when interpreting the findings. Although the sample size (*n* = 253) was sufficient for descriptive and exploratory analyses, a qualitative study is needed to provide deeper insight into and explanation of the findings from the quantitative approach. This could be strengthened by increasing the study site to include some of the district or regional hospitals within the province to capture more perspectives into the influences of depressive symptoms. The cross-sectional design restricts the ability to establish causal relationships between caregiving burden, socioeconomic factors, and depressive symptoms. The CES-D has demonstrated reliability in South African populations; the 20-item version has not been extensively validated specifically among caregivers, which may affect measurement precision. At the same time, reliance on self-reported measures may introduce recall or social desirability bias, potentially affecting the accuracy of reported outcomes. Purposive sampling limits the generalisability of findings because the sample is not randomly selected and is chosen based on specific criteria, potentially making it unrepresentative of the broader population. Because the sample is chosen intentionally, the results are typically applicable only to the particular group studied (caregivers) and cannot be confidently extended to other groups or to the entire population. Potential confounders, such as caregivers’ physical health and family support networks, were not fully assessed, which could influence depressive outcomes. Future research should employ longitudinal designs to better understand the dynamic relationship between caregiving burden and depressive symptoms over time, expand geographic and demographic representation to enhance generalisability, and investigate protective factors such as social support networks, effective coping strategies, and access to formal caregiver resources.

What we could not identify, and a key lesson learned, was the underlying mechanisms that may explain unexpected patterns in depressive symptoms across socioeconomic strata. Factors such as stigma, cultural expectations, or informal support systems could not be explored because qualitative data were not collected. Consequently, qualitative research is needed to provide a deeper explanation of the findings. These gaps highlight an important insight: inconclusive or partial results can guide future work, including studies with larger stratified samples and mixed-methods designs.

## 6. Conclusions

This study highlights the significant impact of caregiving for visually impaired individuals on caregivers’ mental health, particularly depressive symptoms, and underscores the influence of socioeconomic factors, gender, age, marital status, education, and income. Female and lower-income caregivers, as well as those with lower educational attainment, were disproportionately affected. In contrast, male caregivers faced unique emotional, financial, and physical burdens that were compounded by weaker support networks and underutilization of caregiver resources. The findings emphasise the need for targeted interventions, including psychosocial support, financial assistance, and community-based respite services, that are sensitive to both gender and socioeconomic context. Addressing these disparities is crucial for improving caregiver well-being and ensuring sustainable, high-quality care for visually impaired individuals.

## Figures and Tables

**Table 1:** Characteristics of Family Caregivers’ Sociodemographic and Socioeconomic Status for the Visually Impaired.

	*n* = 253	Female	Male	*p*-Value for Trend	Cramer’s V
Sociodemographic Variables	*n* (%)	*n* (%)	*n* (%)		
Age in years					
22–34	57 (22.5)	31 (17.5)	26 (34.2)	0.003	0.251
35–49	82 (32.4)	60 (33.9)	22 (28.9)		
50–59	46 (18.2)	33 (18.6)	13 (17.1)		
60–69	36 (14.2)	33 (18.6)	3 (3.9)		
70+	32 (12.7)	20 (11.3)	12 (15.8)		
Marital status					
Married	99 (39.1)	65 (36.7)	34 (44.7)	0.121	0.152
Never married/cohabited	133 (52.7)	96 (54.2)	37 (48.7)		
Divorced with a partner	12 (0.5)	7 (4.0)	5 (6.6)		
Partner deceased	9 (0.4)	9 (5.1)	0 (0.0)		
Level of education					
No education or primary	30 (11.9)	15 (8.5)	15 (19.7)	0.034	0.164
Secondary	132 (52.2)	94 (53.1)	38 (50.0)		
Tertiary	91 (35.9)	68 (38.4)	23 (30.3)		
Employment status					
Employed	95 (37.6)	45 (25.2)	50 (65.8)	<0.001	0.382
Unemployed	158 (62.5)	132 (83.5)	26 (16.5)		
Income in rands					
16,221.87	32 (12.6)	16 (9.0)	16 (21.1)	<0.001	0.374
8111.24–16,221.86	24 (9.5)	9 (5.1)	15 (19.7)		
6067.90–8111.23	9 (3.6)	4 (2.3)	5 (6.6)		
4055.52–6067.89	13 (5.1)	6 (3.4)	7 (9.2)		
2432.98–4055.51	23 (9.1)	19 (10.7)	4 (5.3)		
812.10–2432.97	67 (26.5)	52 (29.4)	15 (19.7)		
812.09	85 (33.6)	71 (40.1)	14 (18.4)		
Socioeconomic status					
Upper (I)	12 (4.7)	4 (2.3)	8 (10.5)	<0.001	0.329
Upper Middle (II)	45(17.8)	22 (12.4)	23 (30.3)		
Lower Middle (III)	20(7.9)	11 (6.2)	9 (11.8)		
Upper Lower (IV)	133(52.6)	104 (58.8)	29 (38.2)		
Lower (V)	43 (17.0)	36 (20.3)	7 (9.2)		

**Table 2:** The mean scores for depressive symptoms, along with their standard deviations.

Centre for Epidemiologic Studies Depression Scale (CES-D)			
	Questions	Mean	Std. Deviation
**1.**	I was bothered by things that usually don’t bother me.	0.91	0.868
**2.**	I did not feel like eating; my appetite was poor.	0.77	0.857
**3.**	I felt that I could not shake off the blues, even with help from my family or friends.	0.61	0.850
**4.**	I felt I was just as good as other people.	0.96	1.110
**5.**	I had trouble keeping my mind on what I was doing.	0.98	1.000
**6.**	I felt depressed.	0.88	1.002
**7.**	I felt that everything I did was an effort.	1.28	1.107
**8.**	I felt hopeful about the future.	1.27	1.195
**9.**	I thought my life had been a failure.	0.51	0.829
**10.**	I felt fearful.	0.83	0.986
**11.**	My sleep was restless.	1.00	0.930
**12.**	I was happy.	0.99	1.090
**13.**	I talked less than usual.	0.91	0.960
**14.**	I felt lonely.	0.70	0.911
**15.**	People were unfriendly	0.70	0.959
**16.**	I enjoyed life.	1.03	1.125
**17.**	I had crying spells.	0.86	0.919
**18.**	I felt sad.	0.78	0.877
**19.**	I felt that people disliked me.	0.65	0.925
**20.**	I could not get “going”.	0.55	0.870

**Table 3:** The relationships among demographics, socioeconomic status, and depressive symptoms.

	*n* = 253	Depressive Symptoms				*p*-Value for Trend	Cramers-V
Sociodemographic Variables		Little *n* (%)	Mild *n* (%)	Moderate *n* (%)	Severe *n* (%)		
Age							
22–34 (young adults)	57 (22.5)	10 (17.5)	17 (29.8)	19 (33.3)	11 (19.3)	0.211	0.157
35–49 (adults)	82 (32.4)	12 (14.6)	23 (28.0)	29 (35.4)	18 (22.0)		
50–59 (middle-aged)	46 (18.2)	13 (28.3)	9 (19.6)	9 (19.6)	15 (32.6)		
60–69 (elders)	36 (14.2)	9 (25.0)	10 (27.8)	11 (30.6)	6 (16.7)		
70+ (older groups)	4 (1.6)	10 (31.3)	12 (37.5)	6 (18.8)	4 (12.5)		
Marital status							
Married	99 (39.1)	20 (20.2)	18 (18.2)	34 (34.3)	27 (27.3)	0.058	0.146
Never married/cohabited	133 (52.6)	29 (21.8)	45 (33.8)	36 (27.1)	23 (17.3)		
Divorced with a partner	12 (4.7)	5 (41.7)	4 (33.3)	2 (16.7)	1 (8.3)		
Partner deceased	9 (3.6)	0 (0.0)	4 (44.4)	2 (22.2)	3 (33.3)		
Education level							
No education or primary	30 (11.9)	1 (3.3)	3 (10.0)	10 (33.3)	16 (53.3)	<0.001	0.481
Secondary	132 (52.2)	18 (13.6)	40 (30.3)	45 (34.1)	29 (22.0)		
Tertiary	91 (35.9)	35 (38.5)	28 (30.8)	19 (20.9)	9 (9.9)		
Employment status							
Unemployed	158 (62.5)	51 (32.3)	47 (29.7)	39 (24.7)	21 (13.3)	<0.001	0.398
Employed	95 (37.6)	3 (3.2)	24 (25.3)	35 (36.8)	33 (34.7)		
Income in rands							
>16,221.86	32 (12.6)	0 (0.0)	3 (9.4)	10 (31.3)	3 (59.4)	<0.001	0.757
8111.24–16,221.86	24 (9.5)	0 (0.0)	6 (25.0)	8 (33.3)	10 (41.7)		
6067.90–8111.23	9 (3.6)	0 (0.0)	3 (33.3)	3 (33.3)	3 (33.3)		
4055.52–6067.89	13 (5.1)	2 (15.4)	5 (38.5)	6 (46.2)	0 (0.0)		
2432.98–4055.51	23 (9.1)	1 (4.3)	9 (39.1)	8 (34.8)	5 (21.7)		
812.10–2432.97	67 (26.5)	22 (32.8)	24 (35.8)	14 (20.9)	7 (10.4)		
<812.09	85 (33.6)	29 (34.1)	21 (24.7)	25 (29.4)	10 (11.8)		

**Table 4:** The odds ratios for various demographics and socioeconomic status with depressive symptoms.

Variable	Little	Mild to Moderate	Moderate to Severe	Severe
Gender				
Female	Ref	Ref	Ref	Ref
Male	0.3 (0.12–0.65) ^&^	1.4 (0.78–2.50) ^^^	1.5 (0.86–2.71) ^^^	1.1 (0.57–2.09) ^^^
Age				
22–34	Ref	Ref	Ref	Ref
35–49	1.4 (0.64–2.91) ^^^	0.89 (0.47–1.71) ^^^	0.8 (0.41–1.47) ^^^	1.2 (0.56–2.46) ^^^
50–59	1.3 (1.0–1.58) ^@^	1.1 (0.85–1.29) ^^^	0.8 (0.67–1.03) ^^^	1.0 (0.73–1.17) ^^^
Marital status				
Married	Ref	Ref	Ref	Ref
Never married	1.1 (0.58–2.09) ^^^	2.3 (1.23–4.30) ^@^	0.7 (0.40–1.25) ^^^	0.6 (0.30–1.05) ^^^
Divorced	2.8 (0.81–9.83) ^^^	2.3 (0.61–8.30) ^^^	0.4 (0.79–1.84) ^^^	0.24 (0.30–1.97) ^^^
Widow/wee	-	3.6 (0.88–14.75) ^^^	0.6 (0.11–2.78) ^^^	1.3 (0.31–5.71) ^^^
Education				
Tertiary	Ref	Ref	Ref	Ref
Secondary	4.6 (0.59–35.73) ^^^	3.9 (1.12–13.65) ^@^	1.03 (0.45–2.40) ^^^	0.3 (0.10–0.56) *
Primary	18.1 (2.36–139.07) ^@^	4 (1.11–14.29) ^@^	0.53 (0.21–1.31) ^^^	0.1 (0.36–0.26) ^#^
Income				
16,221.87	Ref	Ref	Ref	Ref
8111.24–16,221.86	-	1.0 (0.36–2.89) ^^^	1.2 (0.46–3.16) ^^^	5.4 (1.88–15.25) ^&^
6067.90–8111.23	-	1.5 (0.35–6.63) ^^^	1.2 (0.28–5.18) ^^^	3.8 (0.81–17.4) ^^^
4055.52–6067.89	0.4 (0.73–1.69) ^^^	1.9 (0.56–6.46) ^^^	2.1 (0.63–6.73) ^^^	-
2432.98–4055.51	0.9 (0.11–0.68) ^@^	2.0 (0.74–5.18) ^^^	1.3 (0.48–3.40) ^^^	2.1 (0.63–6.85) ^^^
812.10–2432.97	0.9 (0.48–1.86) ^^^	1.7 (0.84–3.43) ^^^	0.6 (0.30–1.34) ^^^	0.9 (0.31–2.44) ^^^

^#^ *p* < 0.001, ^^^ *p* > 0.050, * *p* = 0.001, ^&^ *p* > 0.001, ^@^ *p* < 0.050.

## Data Availability

The data of this study are available from the corresponding author, D.G.M., upon request. Any inquiries can be directed to the corresponding author.
